# The silent influence: experimental validation of cultural unconscious and its psychological impact through Chinese archetypal imagery

**DOI:** 10.3389/fpsyg.2025.1657224

**Published:** 2025-11-14

**Authors:** Wenlong Wang, Heyong Shen, Xin Zhang, Langyi Wen

**Affiliations:** 1 Psychological Counseling Center, Guangdong University of Finance and Economics, Guangzhou, China; 2 Institute of Analytical Psychology, City University of Macau, Macau, China; 3 School of Public Administration, South China University of Technology, Guangzhou, China; 4 School of Big Data and Artificial Intelligence, Guangdong University of Finance and Economics, Guangzhou, China

**Keywords:** cultural unconscious, Chinese cultural archetypal imagery, empirical study, unconscious influence, archetypes and behavior

## Abstract

This paper empirically confirms Henderson's theory of cultural unconscious which is a psychic layer mediating between collective unconscious and the personal unconscious. We particularly tested the effect of high-valence Chinese archetypal imagery on cognition. A multi-method experimental design was applied to three studies to demonstrate unconscious activation: (1) Reaction Time task proved faster cognitive processing to the exemplary archetypal images than the nonrepresentative ones (study1); (2) Supraliminal Priming followed by a situational choice task proved that there was a significant, unconscious bias of behavioral choices that followed the symbolic meanings of the images (study2); (3) Rigorous Subliminal Priming demonstrated that cultural unconscious could be activated to influence semantic alignment in a word-choice task, even outside of conscious awareness (study3). These data provide solid, quantitative proof that the cultural unconscious is an operatively effective psychological structure, bridging depth psychology concepts with contemporary cognitive science.

## 1 Introduction

### 1.1 The theoretical understanding of the unconscious mind

The concept of the personal unconscious was initially developed by Sigmund Freud in the understanding of the repressed, dynamic, personal realm that limits the experiences of an individual (Freud, 1915). Developing this model, Carl G. Jung added the layer of the collective unconscious to it as a deeper inherited psychic substratum which received archetypes, the primordial, universal templates of images and behaviors that all humans share (Jung, 1968; West, 2011).

In order to mediate the process between the universal collective and the individual-personal, Joseph L. Henderson came up with the concept of cultural unconscious (Henderson, 1990). This idea states a psychic layer, which structures the demonstration of universal archetypes in the form of historical narratives, symbols, and myths of a particular culture. It exists as a group, inherited, but culturally programmed entity, an abstract way of bridging between the model of Jung and the model of Freud. This concept has been able to be clinically pursued with the concept of Cultural Complexes, collective, feeling psychic organization, which forms part of group behavior and perception (Weisstub and Weisstub, 2004).

### 1.2 Contemporary research on culture and non-conscious cognition

In recent decades, empirical cultural psychology has provided robust support for the dynamic influence of cultural mental structures on fundamental cognitive processes, often utilizing cultural priming techniques. This approach views culture not as a fixed trait but as a dynamic mental schema that can be temporarily activated (Markus and Kitayama, 1991).

The established cultural priming literature demonstrates that activating cultural mindsets, such as individualism vs. collectivism, systematically modulates self-concept, relational assumptions, and values (Oyserman and Lee, 2008). This has been shown to influence basic perception and judgment, with collectivistic primes promoting holistic thinking (contextual focus) and individualistic primes promoting analytic thinking (object focus) (Nisbett et al., 2001). More specifically, research has linked subliminal cultural priming to modulations of the self-bias effect in perceptual matching and the activation of specific neural circuits related to self-processing (Han and Northoff, 2020; Jiang and Sui, 2022). Furthermore, researchers have investigated the role of culture in shaping the very structure of non-conscious cognition and perception, urging for integration between neuroscience and cultural psychology (Yakushko et al., 2016; Wang, 2016).

### 1.3 The research gap: lack of empirical validation for archetypal structures

Although the evidence concerning dynamic cultural mindsets is very strong, there is serious research gap: there is no direct multi-method experimental verification of the cultural unconscious of Henderson in the form of its main ingredient, i.e., archetypal imagery. The literature available to date on priming is more abstract based on its cognitive dimensions in contrast to its deep, symbolic, and historical based content, which constitutes the cultural unconscious. So far, no research on how culture-specific and high-valence archetypal images are unconsciously processed and their influence on the basic thinking is done in a systematic way. Sealing this divide is crucial toward incorporating the depth psychological ideas of the cultural unconscious in the evidence based paradigm of cognitive and cultural psychology.

### 1.4 Focus of the current study and hypotheses

In order to bridge such theoretically and empirically significant gap, the current research employs the Chinese archetypal images (e.g., the Dragon, Tai Chi) as an experimentally operationalized exemplification of the cultural unconscious. We hypothesize such images of high value, are powerful priming stimuli and the deep cultural unconscious structure is activated to influence the immediate cognition and behavior of an individual. This study uses a combination of explicit association test, contextual (supraliminal) priming test, and subliminal priming test to explore the depth and breadth of this effect.

According to the theory of Henderson and the processes of psychological priming, we can put forward the following hypotheses:

H1 (Explicit Association): The explicit correlation of words and representative archetypal imagery will provide the subjects with the group of people with a relevant cultural background with significantly faster reaction times as opposed to the explicit correlation of words and non-representative imagery, proving that cognitive processing is facilitated.

H2 (Contextual Priming): When exposed to representative archetypal imagery (supraliminal priming), the participants will unknowingly bias their future behavioral decisions by selecting choices that align with the symbolic meaning of the image.

H3 (Subliminal Priming): Even when archetypal imagery is exposed to the participants below the level of the conscious awareness (subliminal priming), the cognitive and semantic decisions of the participants are going to be affected significantly.

## 2 Study 1: cultural archetypal imagery association test

### 2.1 Methods

#### 2.1.1 Design

Study 1 employed a within-subjects design, selecting participants of Han ethnicity from a Chinese cultural background. The participants were tasked with generating associative words for 50 representative images of Chinese cultural archetypes and 50 non-representative images, which they entered as their responses.

The experiment measured participants' reaction times and the associative words they provided. By comparing the differences in reaction times and the content of associations between the representative and non-representative images, this study aimed to demonstrate the influence of the cultural unconscious on individuals.

#### 2.1.2 Materials

The cultural unconscious and cultural archetypes are abstract concepts, primarily manifested through archetypal imagery. Consequently, the experimental materials consisted of images representing cultural archetypes, which symbolized the cultural unconscious.

To ensure the representativeness of the Chinese cultural archetype images for the experimental group and the non-representativeness of the control group images, the researchers initially selected 118 potential images of Chinese cultural archetypes and 96 potential non-representative images.

A total of 230 participants of Han ethnicity were recruited, including 103 males and 127 females, with a mean age of 20.68 years. All the participants had an undergraduate education and no religious affiliation.

The participants evaluated the representativeness of all 214 images via a 10-point scale, where 0 indicated no representativeness and 10 indicated the highest representativeness. The evaluation was conducted through an online questionnaire, with the images presented in a random order.

On the basis of the representativeness ratings of the images and their categories (e.g., humans, animals, plants, architecture, natural landscapes, objects, and culture), 50 images representing Chinese cultural archetypes and 50 non-representative images were selected.

To verify the difference between the Chinese cultural archetype representative images and non-representative images, an independent samples *t*- test was conducted on the representativeness ratings of the two categories. The results revealed a significant difference (*t* = 56.47, *p* = 0.000) between the Chinese cultural archetypal representative images (*M* = 8.36, *SD* = 0.65) and non-representative images (*M* = 1.40, *SD* = 0.58), indicating that the material selection was scientifically sound.

To minimize the impact of color and size variations on the experimental results, all images in this study were converted to black and white, uniformly scaled to maintain proportionality, and centered within the same background (size: 47.6 cm × 26.8 cm).

#### 2.1.3 Participants

To minimize the impact of inter-subject variability on the experimental results, 46 participants were selected for this study (22 males, 24 females; mean age = 20.72 years). All participants were of Han ethnicity, had undergraduate education, reported no religious affiliations, were right-handed, had normal or corrected-to-normal vision, and had no physical disabilities that could interfere with the experimental procedures. Informed consent was obtained from all participants, who were compensated with cash.

#### 2.1.4 Procedure

Experiment 1 used E-Prime 3.0 to design the experimental program, which was executed on a Tsinghua Tongfang H110-4S computer with a 21.7-inch display screen, an aspect ratio of 16:9, and a resolution of 1,920 × 1,080 pixels.

Each participant was tested individually. The experimenter first explained the procedure to the participant while ensuring that the true purpose of the experiment remained undisclosed.

**Figure 1:**
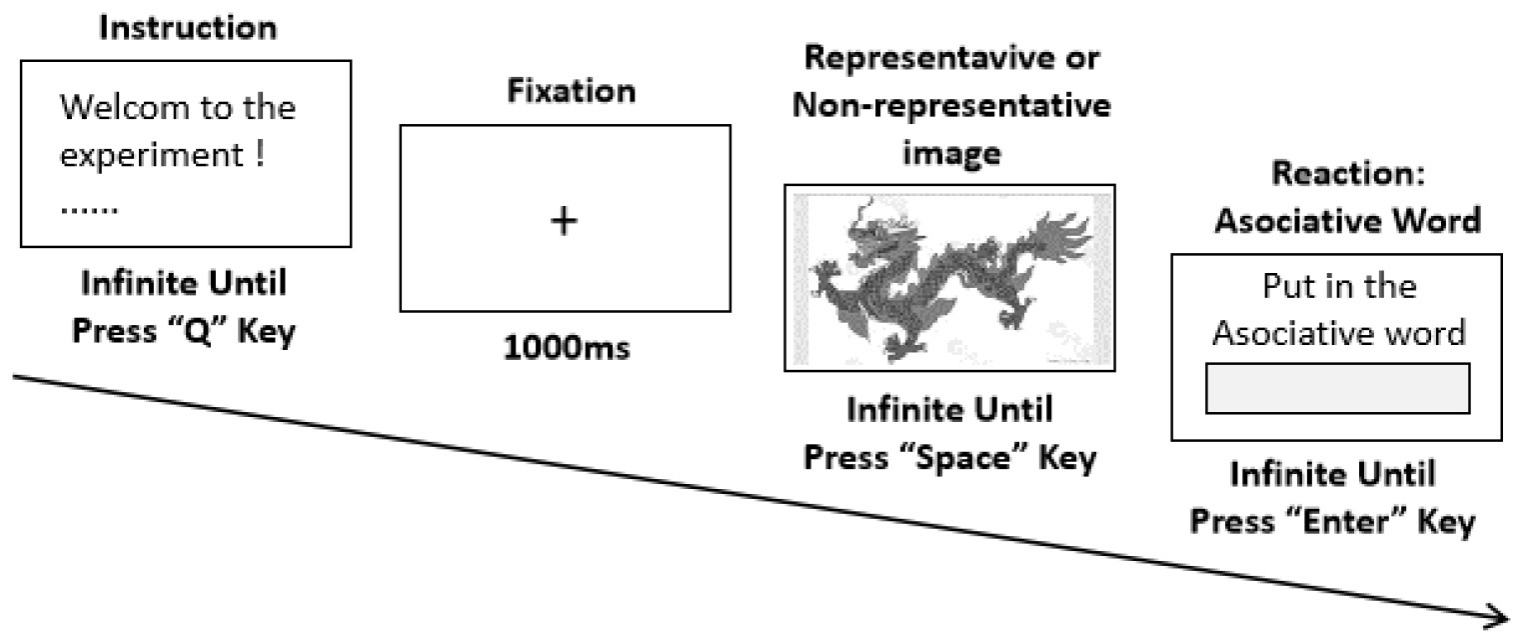
Experimental procedure of the cultural archetypal imagery association test.

The experimental procedure is illustrated in Figure 1.

The computer screen initially displayed the instructions, and the participants were instructed to press the “Q” key to begin. A fixation cross (“+”), RGB (255, 0, 0), with a font size of 34, appeared at the center of the screen for 1,000 ms. Afterward, one of the images from the experimental materials—either a representative or non-representative Chinese cultural archetype image—was randomly presented on the full screen. The image size was 47.6 cm × 26.8 cm, and the display time was set to infinity.

Once the participants saw the image, they were instructed to immediately begin their associations. Prior to the experiment, the researcher reminded the participants to associate with the image rather than name it. Upon the first associative word appearing in their mind, the participants pressed the spacebar, and a text box appeared on the screen. They then entered their associative words (in simplified Chinese) and pressed the “Enter” key to proceed to the next trial.

Experiment 1 presented 50 representative and 50 non-representative images of Chinese cultural archetypes in randomized order, totaling 100 trials. The participants were asked to input associative words for each image, and the experiment concluded when all trials were completed. The screen then displayed the end note of the experiment.

### 2.2 Results

The experimental data were aggregated via E-Merge 3 and subsequently exported to SPSS 22 for further processing and analysis. To ensure the scientific validity of the data, the 4600 entries collected from 46 participants were first organized. The maximum and minimum reaction times for each participant were removed, along with the maximum and minimum reaction times for each image. Additionally, data with excessively long reaction times (> 10,000 ms) caused by participant distractions were excluded. After these adjustments, the dataset consisted of 2,213 valid responses for representative Chinese cultural archetype images and 2,196 valid responses for non-representative images.

**Figure 2:**
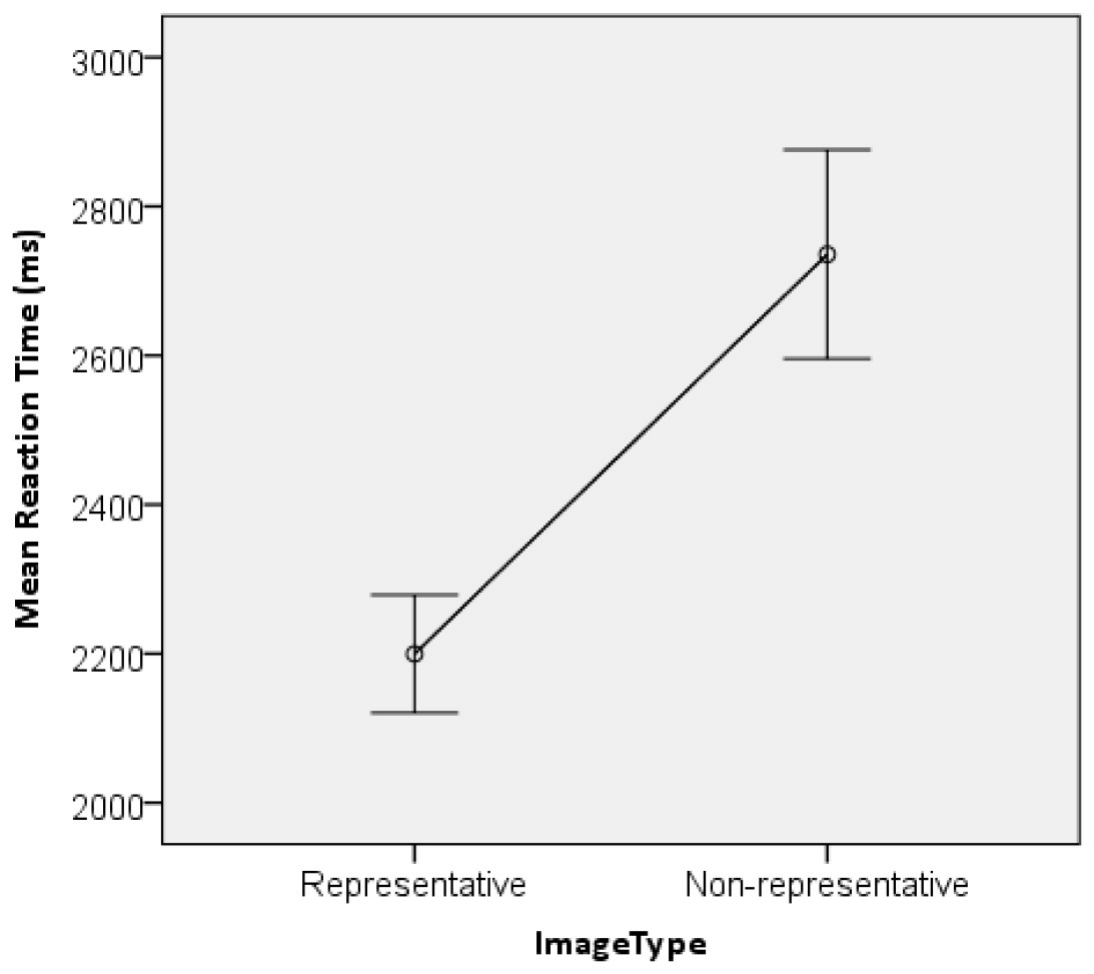
Differences in the reaction times of Chinese cultural archetypal representative images and nonrepresentative images.

To examine differences in unconscious-level responses to representative vs. non-representative Chinese cultural archetype images, an independent samples *t*-test was conducted on participants' reaction times (Figure 2).

Statistical analysis revealed that the average reaction time for representative Chinese cultural archetype images was 2,199.7 ms, which was significantly shorter than the average reaction time for non-representative images (2735.7 ms), *t* = 42.85, *p* < 0.001. This finding indicates that Chinese cultural background facilitates the unconscious processing of representative cultural information among participants.

### 2.3 Discussion

Study 1 set to offer the first empirical signs of the structural presence of the cultural unconscious by gauging the automatic cognitive availability of its main contents: culturally representative archetypal imagery. The results that the participants showed dramatically less reaction time (RTs) when matched with words with representative Chinese archetypal images and not-representative images indicate that the core hypothesis was correct and is in agreement with the accepted principles of implicit cognition.

The reason why the observed faster RT was more likely is that representative archetypal imagery has a higher chronologically established level of pre-activation or accessibility to the Chinese cultural psyche. This finding is a first-hand empirical evidence of the theoretical frameworks advanced by both Jung and Henderson.

The high accessibility can be explained as the enduring imprint of Jung's collective archetypes as they are filtered and specified by Henderson's cultural unconscious (Jung, 1968; Henderson, 1990). Semantic network related to such symbols as the Dragon is installed in the collective mind because of the centuries of cultural repetitions they have been going through and are processed by the viewer at an automatic and rapid pace. The structural organization of the cultural unconscious as a collective layer is a validated effect, which facilitates culturally relevant cognition (Hong et al., 2000).

The mechanism aligns with the results achieved in implicit cognition and semantic priming (Greenwald and Banaji, 1995). The standard semantic priming paradigms requires a faster response to a target word following exposure to a semantically related prime, the paradigm assumes that the semantic priming activated the target word semantic node. In this case, the archetypal image is considered a chronic and pre-activated prime which means that the whole cognitive system underlying the cultural meaning of the image is highly ready.

Moreover, this observation is in line with the cultural neuroscience studies that reveal that culture is not merely a high-level attitude but a dynamic mental structure that regulates neural processing. Cultural priming studies have shown that by engaging certain cultural mentalization, it is possible to modulate the pace and distribution of neural activity (Hedden et al., 2008), implying that the collective salience herein is based on incredibly efficient culture-selective neural mechanisms (Chiao, 2009).

Although this shorter RT might be explained by general familiarity effects, or even, by cultural salience, they are not regarded as a direct rival of one another, but as the mechanisms of cultural unconscious preservation and expression. The archetypes of the cultural unconscious are the most commonly familiar and the most salient cultural products by their very nature as they have been discursively spread and supported across generations. Hence, the empirical measure of the high salience simply reflects the psycho-cultural unconscious in action, supporting the notion that it guides even the most basic cognitive processes (Nisbett et al., 2001).

In the practical sense, there are significant implications in validation of the cognitive accessibility of the cultural unconscious. Learning how sensitive to the environment a client has incorporated these deep archetypal structures can be useful in cultural accommodation of treatment in areas such as psychotherapy (Sue and Sue, 2013). As an example, such positive unconsciously positive valence of a symbol as the Dragon can be used therapeutically. Their effective processing in the sphere of education and communication implies that they are highly effective and fast tools of passing the cultural identity and shared values.

## 3 Study 2: priming effect of Chinese cultural archetypal imagery

### 3.1 Methods

#### 3.1.1 Design

The priming effect paradigm is a crucial framework for studying unconscious processes. To investigate the priming effect of cultural unconsciousness, Study 2 adopted a between-subjects design with experimental and control groups. For participants in the experimental group, representative images of Chinese cultural archetypes were presented as priming stimuli, followed by scenario-based questions (target stimuli) requiring participants to make a choice. In contrast, participants in the control group proceeded directly to the same scenario-based questions without viewing the priming images.

Each scenario-based question included two options: one related to the symbolic meaning of the priming image and the other unrelated. The experiment recorded and compared the frequency and proportion of responses consistent with the symbolic meaning of the priming image between the experimental and control groups. This design aimed to validate the existence of symbolic information priming effects of cultural unconsciousness and their influence on individuals' cognition and behavior.

#### 3.1.2 Materials

##### 3.1.2.1 Priming stimuli materials

Representative images of Chinese cultural archetypes were used as materials to represent the cultural unconscious. The same set of 50 processed representative images of Chinese cultural archetypes from Study 1 was used as the priming stimulus materials.

##### 3.1.2.2 Probe stimuli materials

To correspond with the symbolic meanings of the 50 representative images of Chinese cultural archetypes used as priming stimuli, 50 situational multiple-choice questions were designed as probe stimuli (Table 1). Each question contained two options: one related to the symbolic meaning of the corresponding image, and the other unrelated. The options were assigned response keys labeled “F” and “J”. To avoid the confounding influence of response key assignments on experimental outcomes, 25 related options and 25 unrelated options were assigned to each key (F or J). Additionally, the presentation of each priming stimulus and its corresponding probe stimulus was randomized during the experiment.

**Table 1:** Examples of probe stimuli materials in study 2.

**Priming image**	**Symbolic meaning**	**Probing question**	**Options**	**Consistent option**
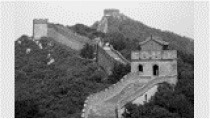 The Great Wall	Stubborn, unyielding	Xiao Zhang felt very tired halfway up the mountain, do you think he will?	F. Continue to climb to the top of the mountain J. Switch to a ropeway up the mountain	F
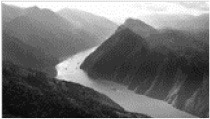 Yangtze River	Flowing forever	Spring buds break through the ground, and after a rainstorm, do you think it will?	F. Wither and die J. Thrive	J
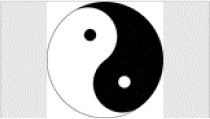 Tai Chi	Chinese philosophy	Xiao Zhou needs to take a public class in college, what do you think he will choose?	F. Philosophy J. Economic	F
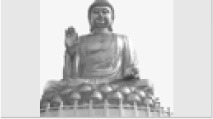 Buddha	Enlightenment, quiet	Xiao Zhao had a quarrel with a friend today, do you think he will?	F. Unpleasant for the whole day J. Calm down soon	J
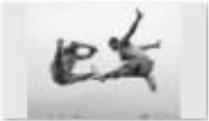 Kung Fu	Chivalrous and righteous	Xiao Wang saw a person stealing a mobile phone when he was taking the bus, do you think he would?	F. Regardless of it J. Stand forward and stop it	J
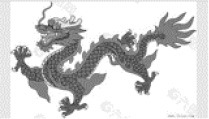 Dragon	Totem, royal	Xiao Zhang is watching a TV series, what do you think he is watching?	F. Urban TV series J. Palace TV series	J

#### 3.1.3 Participants

Study 2 included 90 participants in the experimental group (42 males, 48 females; mean age = 20.73 years) and 108 participants in the control group (51 males, 57 females; mean age = 20.65 years). There was no significant difference in age between the experimental and control groups (*t* = −0.32, *p* = 0.75). To control for potential confounding variables, such as cultural background, religious beliefs, and physical conditions, all 198 participants were Han Chinese, had no religious affiliations, were right-handed, and had normal or corrected-to-normal vision.

#### 3.1.4 Procedure

**Figure 3:**
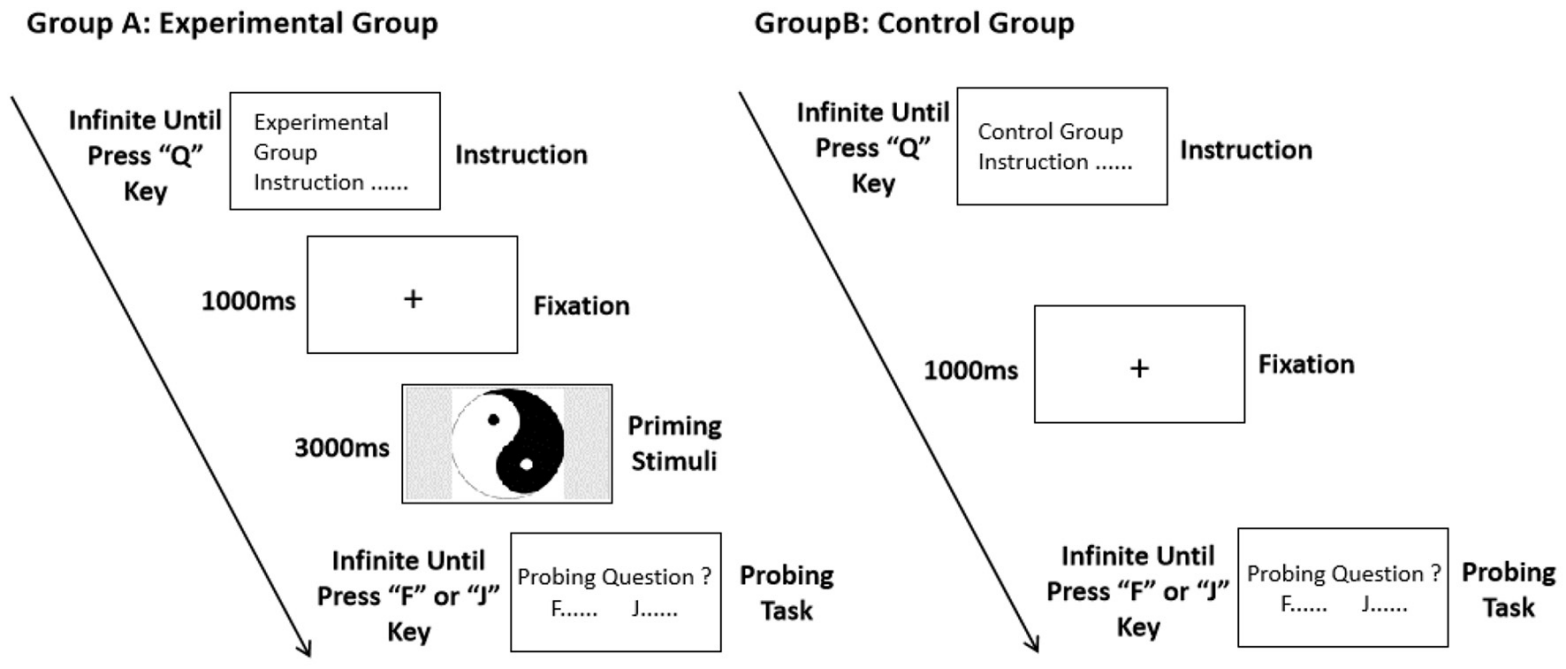
Experimental procedures of experimental group and control group in study 2.

Study 2 was designed and executed via E-Prime 3.0, with the same equipment configuration as in Study 1. The experimental procedure is depicted in Figure 3.

At the beginning of the experiment, the participants in the experimental group were presented with general instructions on the computer screen, which remained visible until they pressed the “Q” key to begin. Each trial started with the display of a fixation point (“+”) in the center of the screen for 1,000 ms. Next, an image representing a Chinese cultural archetype was presented as the priming stimulus for 3,000 ms. Afterward, a context-based multiple-choice question (probe task) appeared on the screen. The participants were instructed to respond by pressing either the “F” key or the “J” key, with the task remaining visible until a response was made. After responding, the program advanced to the next trial.

The experiment consisted of 50 trials in total, each with one Chinese cultural archetype image as the priming stimulus and one corresponding probe task, all presented in random order. To balance the responses, 25 consistent and 25 inconsistent options in the probe tasks were assigned to the “F” and “J” keys. The experiment concluded once the participants had completed all 50 trials.

For the control group, the participants did not view the priming stimulus images. Instead, they directly responded to context-based multiple-choice questions. The remaining experimental procedures were identical to those used for the experimental group.

### 3.2 Results

The data from both the experimental and control groups were first aggregated via E-Merge 3, and subsequently imported into SPSS 22 for further analysis. The frequency and proportion of participants' responses to the probe tasks, which aligned with the symbolic meanings of the priming stimuli, were calculated. The data were then compiled for each individual priming stimulus and its corresponding probe task, yielding a total of 50 data sets. Chi-square tests were conducted on these 50 sets to compare response patterns between the experimental and control groups, thereby testing the priming effect of cultural unconsciousness.

For example, consider the analysis of results for the Chinese cultural archetypal image “firecrackers”. The corresponding probe task was as follows: “Question: In the early morning, Xiao Yang left home as usual. What do you think will happen to him today? Options: F. A regular day; J. Something good happens.” Option J is consistent with the symbolic meaning of firecrackers, which is “celebration”. The experimental results revealed that, among the 90 participants in the experimental group, 57 selected a consistent response (63.33%), whereas 26 of the 108 control group participants selected a consistent response (24.07%). A chi-square test revealed that the experimental group's consistency of choice was significantly higher than that of the control group (χ^2^ = 31.08, *p* < 0.001).

Following this approach, chi-square tests were conducted on all 50 sets of experimental data corresponding to the symbolic priming effects of Chinese cultural archetypal images. The statistical results are presented in Table 2.

**Table 2:** Chi-square tests of the priming effect of Chinese cultural archetypal imagery (10 examples of 50).

**Priming image**	**Experimental group (consistent frequency)**	**Control group (consistent frequency)**	***χ^2^***	***P***
Couplets	83.33%	70.37%	4.56	0.033
Terracotta	65.56%	47.22%	6.68	0.010
Buddha	52.22%	34.26%	6.49	0.011
Kong Fu	85.56%	70.37%	6.45	0.011
Dragon	71.11%	37.96%	21.66	0.000
Wukong	81.11%	65.74%	5.85	0.016
Tai Chi	62.22%	45.37%	5.60	0.018
The Great Wall	71.11%	52.78%	6.94	0.008
Imperial Palace	64.44%	43.52%	9.36	0.002
Oracle	78.89%	39.81%	30.69	0.000

The statistical analysis revealed that, for the 50 selected Chinese cultural archetypal images used as priming stimuli, participants in the experimental group consistently reported significantly higher frequencies of choosing options aligned with the symbolic meanings of the images in the situational multiple-choice tasks than did those in the control group, which did not view the priming stimuli. Among these results, 19 items reached a significance level of *p* < 0.05, 16 items reached *p* < 0.01, and 15 items reached *p* < 0.001.

### 3.3 Discussion

Study 2 showed that supraliminal exposure to culturally representative archetypal imagery has a significant biasing effect on the subsequent behavioral decisions, which presents direct evidence of how the cultural unconscious works in making decisions. The fact that the experimental group chose the option that corresponded to the symbolic meanings of the images at a statistically significant rate more than the control group confirms the hypothesis that the cultural unconscious is a functional psychic structure that can prime behavior unconsciously.

This outcome goes beyond the result of Study 1 which revealed only cognitive accessibility (RT) to offer functional causality. The short, aware perception of the archetypal image that is a very condensed version of cultural meaning was all that would trigger the relevant Cultural Complex (Abramovitch, 2007). This triggering served as an unconscious instruction, confirming Henderson's model: the cultural unconscious serves as a psychic energy source, organizing and directing human behavior within a culturally appropriate system, which does not have to think consciously.

This result aligns with the already documented implications of behavioral priming in implicit cognition (Greenwald and Lai, 2020). When a prime activates a concept, that activated mindset can subsequently apply to other related tasks (the situational choice). But, in contrast to generalized priming, the present study shows that the cultural unconscious is aroused by highly enduring symbolic content, and proves the existence of a specialized form of implicit processing based on centuries of cultural sedimentation.

The identified change in behavior also stays consistent with the studies of the cultural neuroscience that may presuppose different neural networks to be recruited based on culturally salient stimuli that involve processing social cognition (Chiao, 2009). The archetypal image, as a prime cultural icon, must have activated areas of the brain related to social norm and value-based judgments swiftly and without conscious awareness, effectively putting cultural meaning into a preference to act.

The depicted effectiveness of archetypal images in determining the choice of behavior has crucial practical implications in a clinical context. The culturally adjusted psychotherapy depends heavily on recognizing a powerful yet unconscious impact of cultural symbols in a clinical context (Tummala-Narra, 2015). The therapeutic interventions may help more effectively if they consider, rather than ignore, the client's cultural achetypes, which can help integrating these unconscious dynamics into conscious life.

In the context of education and public health promotion, the findings reveal that high-valence cultural archetypes can be an effective, unobtrusive, non-coercive instrument in promoting the desired behavior or inculcating the desired cultural values by circumventing conscious resistance, and have direct impact on collective, unconscious drives.

## 4 Study3: subliminal priming effect of Chinese cultural archetypal imagery

### 4.1 Methods

#### 4.1.1 Design

The subliminal priming paradigm is widely employed in the study of unconscious psychological processes. To investigate the subliminal priming effect of cultural unconsciousness, Study 3 utilized a between-subjects design, consisting of an experimental group and a control group. In the experimental group, the participants were first exposed to Chinese cultural archetypal images as subliminal priming stimuli, followed by a choice task involving two words as detection stimuli. In contrast, participants in the control group were not exposed to the priming images and directly chose between the same pair of word stimuli used in the experimental group.

In the detection task, one word was semantically related to the symbolic meaning of the primed image, whereas the other word was unrelated. The experiment then recorded and compared the frequency and proportion of participants' selections that matched the symbolic meaning of the primed image between the experimental and control groups. This comparison aimed to confirm the presence of a subliminal priming effect on the basis of cultural unconscious information.

#### 4.1.2 Materials

*Masking Stimulus Materials*. The experiment utilized a gray-black opaque grid image as the masking stimulus, with a resolution of 1,280 × 720 pixels and a size of 47.6 cm by 26.8 cm. The masking stimulus was presented both before and after the priming image.

*Priming Stimulus Materials*. The priming stimulus materials consisted of 50 representative Chinese cultural archetypal images, similar to those used in Study 2, all processed in the same manner.

*Detection Stimulus Materials*. For each of the 50 Chinese cultural archetypal images, 50 word-choice questions were designed to correspond to their typical symbolic meanings. Each question included two options: one was semantically related to the symbolic meaning of the corresponding image, and the other was unrelated. The response keys were labeled “F” and “J.” To control for potential bias in the keypress responses, the semantically related and unrelated options were randomly assigned to the “F” and “J” keys in 25 instances each. Additionally, each pair of priming and detection stimuli was randomly presented during the experiment.

The 50 representative Chinese cultural archetypal images and their corresponding options are listed below (Table 3).

**Table 3:** Examples of probe stimuli materials of study 3.

**Priming image**	**Symbolic meaning**	**Probing options**	**Consistent option**	
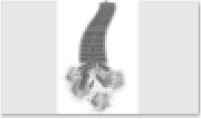 Fireworks	Joyful	F. Command	J. Celebrate	J
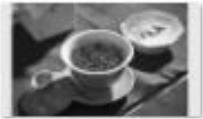 Tea	Indifferent to fame	F. Delicate	J. Gloomy	F
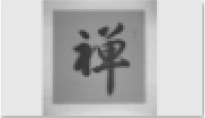 Zen	Calm	F. Running	J. Meditate	J
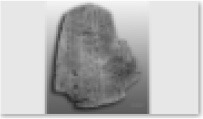 Oracle	Ancient culture	F. Ancient	J. Fresh	F
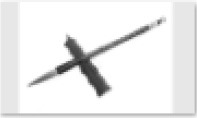 Chinese Brush	Traditional calligraphy	F. Morden	J. Classic	J
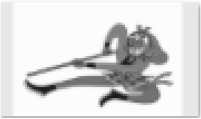 Wukong	Subdue demons	F. Hero	J. Boss	F

#### 4.1.3 Subjects

Study 3 included 51 participants in the experimental group (24 males and 27 females), with a mean age of 20.12 years. The control group comprised 101 participants (47 males and 54 females), with a mean age of 20.27 years. There was no significant difference in age between the experimental and control groups (*t* = −0.94, *p* = 0.35).

To control for potential confounding factors such as cultural background, religious beliefs, and physical conditions, all 152 participants (from both groups) were of Han ethnicity, had no religious affiliation, were right-handed, and had normal or corrected-to-normal vision.

#### 4.1.4 Procedure

**Figure 4:**
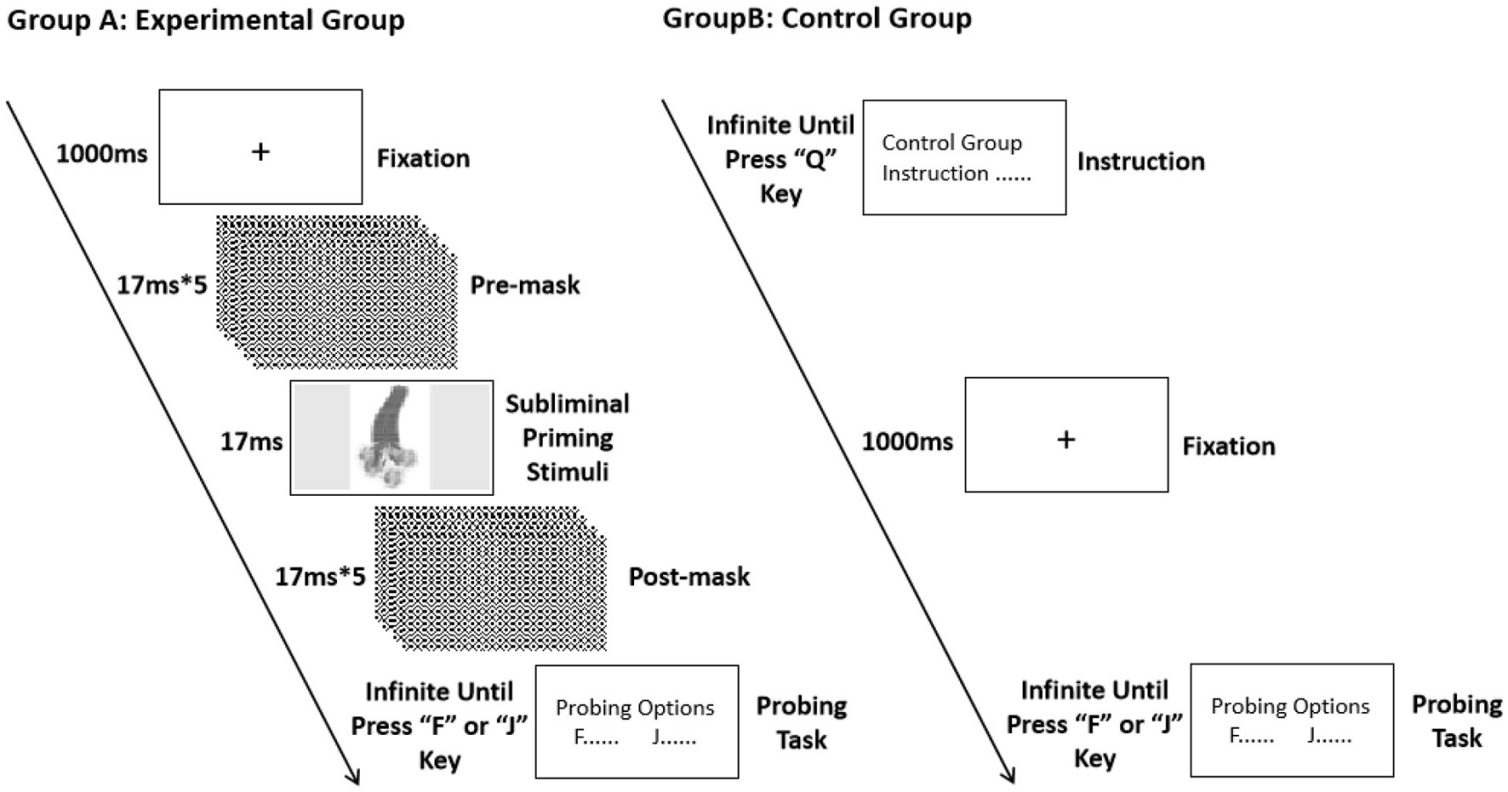
Experimental procedures of the experimental group and control group in study 3.

Study 3 utilized E-Prime 3.0 for designing and executing the experimental procedures. The experimental design is illustrated in Figure 4.

At the beginning of the experiment, general instructions were displayed on the screen with no time limit for reading. After the participants had read and understood the instructions, they initiated the experiment by pressing the “Q” key.

Each trial began with a fixation point (“+”) appearing at the center of the screen for 1,000 ms. To ensure the subliminal presentation of the priming stimuli, five premasking images were shown sequentially, each for 17 ms, for a total of 85 ms. Following premasking, a Chinese cultural archetypal image was presented as the subliminal priming stimulus. The duration of the priming stimulus was set to 17 ms, in line with previous studies (Kiesel et al., 2008), to ensure subliminal exposure.

After the subliminal priming stimulus, five postmasking images were presented sequentially, each for 17 ms, adding up to another 85 ms. A word-choice detection task was subsequently presented, with the display remaining until the participant made a response. The participants were instructed to respond on the basis of their first impression by pressing either the “F” or “J” key.

The experiment included 50 trials, with 50 representative Chinese cultural archetypal images as subliminal priming stimuli and 50 corresponding word-choice questions as detection tasks. The pairs of priming stimuli and detection tasks were presented in random order. For each detection task, the consistent option corresponding to the priming stimulus was equally divided between the “F” and “J” keys, with 25 instances for each.

Following the experiment, the participants were asked whether they had noticed the subliminal priming images during the task. All the participants reported being unaware of the presentation of the priming stimuli.

In contrast, the control group did not receive any masking or priming stimuli. Instead, participants in the control group directly received the same word-choice detection tasks as did those in the experimental group and made their selections on the basis of their first impression by pressing either the “F” or “J” key.

### 4.2 Results

The data collected from both the experimental and control groups were first summarized via E-merge 3.0 and then imported into SPSS 22 for further analysis. The frequency and percentage of participants' responses in the detection task, which aligned with the symbolic meaning of the subliminal priming stimuli, were calculated. A chi-square test was then conducted on the consistency frequency and percentage data from the 50 sets for both the experimental and control groups. This analysis assessed the impact of the subliminal priming stimuli on the experimental group's responses, confirming the existence of the cultural unconscious subliminal priming effect.

For example, in the experiment involving the subliminal priming of the Chinese cultural archetypal image “Terracotta Warriors”, the detection task provided the following options: “F. Unity” and “J. Independence”. The “F” option was consistent with the symbolic meaning of “unity”, which is associated with the Terracotta Warriors. The results revealed that 76.47% of the participants in the experimental group selected the “F” option, a significantly higher percentage than did the 47.52% of participants in the control group (*p* = 0.001).

Chi-square tests were similarly conducted on the remaining 50 sets of data, with the results presented in Table 4.

**Table 4:** Chi-square tests of the subliminal priming effect of Chinese cultural archetypal imagery (10 examples of 50).

**Priming image**	**Experimental group (consistent frequency)**	**Control group (consistent frequency)**	***χ^2^***	***P***
Fireworks	82.35%	62.38%	6.33	0.012
Terracotta	76.47%	47.52%	11.60	0.001
Confucius	84.31%	59.41%	9.62	0.002
Tea	80.39%	61.39%	5.60	0.018
Zen	66.67%	31.68%	16.88	0.000
Wukong	81.11%	65.74%	5.85	0.016
Panda	94.12%	60.40%	19.00	0.000
Chinese Brush	64.71%	13.86%	41.01	0.000
Chopsticks	94.12%	80.20%	5.11	0.024
Oracle	49.02%	23.76%	9.90	0.002

The statistical analysis indicated that for 49 of the 50 subliminal priming detection tasks, participants in the experimental group were significantly more likely to choose responses consistent with the symbolic meanings of the images than were those in the control group, which was not exposed to the priming stimuli. These findings support the significance of the subliminal priming effect of Chinese cultural archetypal images. Specifically, the chi-square test results revealed that 15 items reached a significance level of *p* < 0.05, 11 items reached *p* < 0.01, and 23 items reached *p* < 0.001.

### 4.3 Discussion

Study 3 is the most stringent empirical experimentation of the cultural unconscious as it shows its effect in circumstances of subliminal priming of stimuli when they are introduced beyond the conscious level. This observation, that the participants were much more prone to picking the words that had the semantically oriented meaning with regards to the symbolic meaning of the speedily masked Chinese archetypal images, lending a considerable weight to the hypothesis that this deep layer of the culture is activated and has a cognitive influence without conscious awareness.

This finding becomes instrumental to support the theory by Henderson (1990) and Jung (1968). Because the stimuli (archetypal images) were shown within a very low time (17 ms) and disguised, the given effect cannot be explained by conscious thought, explicit disposition, or strategy production. Rather, this effect confirms that cultural unconscious is an automatic, pre-conscious, structural system of automatically processing and actively putting to use high-valence symbolic content. The archetypes, in the structures laid out by the cultural unconscious, have enough psychic energy to overcome the limen of consciousness and enter semantic-cognitive processing and are covert but powerful organizers of the mind.

The findings are directly correlated with the most effective subliminal types of semantic priming in cognition psychology (Gawronski and Bodenhausen, 2011). Namely, it indicates that the defining cultural notions related to the archetypal images are broadly rooted in the semantic net. It is such cultural richness that allows the masked prime to trigger the relevant semantic nodes, and influences the next selection in the word task. This result is relevant to the literature on cultural priming because it shows that symbolic content with archetypal qualities (not merely verbal self-construal primes) can be a powerful subliminal activator, implying that there is a deeper effect of culture on semantic organization.

A cultural neuroscience point of view on the subliminal influence suggests that archetypal images stimulate specialized and very effective neural loops. It has been found out that the cultural values do regulate the activities of the brain in areas that are concerned with self-processing and social conduct (Kitayama and Uskul, 2011). These deep cultural structures are being instantiated, according to their immediate, unconscious nature, as automatic, hard-wired cognitive routines, triggered more quickly than conscious perception can be registered by the visual system (Ge et al., 2018).

The implication of the confirmation of cultural archetypes playing a significant role in semantic processing without conscious awareness is significant: (1) In a clinical setting, the potency of a culturally applicable collective symbol (e.g., the application of a traditional story or image) could be used to play a role in psychodynamic work. Since these symbols may bypass conscious defenses and opposition, the findings demonstrate the significance of visual and symbolic culture (e.g., in art, media, and ritual) as a non-linguistic tool of conveying values to a client (Groark, 2019; Hwang, 2001). The communication policy makers and educators can acknowledge such cultural assets as a strong automatic engines of cognitive alignment and social cohesion, and this confirms their preservation and cautious use in cultural communication endeavors.

## 5 Conclusions

This research provides multi-method empirical validation for Joseph L. Henderson's theory of the cultural unconscious, a concept previously lacking quantitative evidence. By using high-valence Chinese archetypal imagery, the study confirmed that this psychic layer is a functionally active organizer of the mind. Three experiments offered converging evidence: first, a Reaction Time Task demonstrated the chronic, high accessibility and faster cognitive processing of these cultural structures. Second, a Supraliminal Priming experiment showed that brief exposure to the imagery unconsciously biased behavioral choices toward the symbols' meaning. Finally, a rigorous Subliminal Priming task proved that the cultural unconscious could be activated to influence semantic processing even outside of conscious awareness, establishing its role as an automatic, pre-conscious system.

Understanding the operational influence of the cultural unconscious carries significant practical implications. The results support the need for Culturally Adapted Interventions in psychology, suggesting that therapeutic work can be more effective by strategically engaging a client's core cultural symbols to bypass conscious resistance and facilitate deeper psychological integration. Furthermore, the demonstrated non-conscious priming power indicates that Strategic Communication and educational efforts can leverage these high-valence images as highly efficient, non-verbal tools to instantly transmit cultural values, reinforce collective identity, and subtly promote desired social behaviors.

Despite these robust findings, the study has clear limitations that define future research directions. The focus solely on Chinese archetypes and participants necessitates cross-cultural validation to confirm whether these effects are universal or specific. Future work should also move beyond behavioral measures by incorporating cultural neuroscience techniques (e.g., fMRI, EEG) to map the neural circuitry and temporal dynamics of archetypal activation. Finally, exploring individual differences like acculturation level is crucial to predict the strength of the cultural unconscious's influence among diverse and bicultural populations.

## Data Availability

The raw data supporting the conclusions of this article will be made available by the authors, without undue reservation.
